# Inhibition of influenza A virus and SARS-CoV-2 infection or co-infection by griffithsin and griffithsin-based bivalent entry inhibitor

**DOI:** 10.1128/mbio.00741-24

**Published:** 2024-04-09

**Authors:** Najing Cao, Yanxing Cai, Xin Huang, Hanxiao Jiang, Ziqi Huang, Lixiao Xing, Lu Lu, Shibo Jiang, Wei Xu

**Affiliations:** 1 Key Laboratory of Medical Molecular Virology (MOE/NHC/CAMS), Shanghai Institute of Infectious Disease and Biosecurity, Shanghai Frontiers Science Center of Pathogenic Microorganisms and Infection, School of Basic Medical Sciences, Shanghai Public Health Clinical Center, Shanghai Medical College, Fudan University, Shanghai, China; 2 Guiyang Maternal and Child Health Care Hospital, Guiyang, Guizhou, China; University of Calgary, Calgary, Canada

**Keywords:** respiratory viruses, influenza A virus, SARS-CoV-2, co-infection, griffithsin

## Abstract

Outbreaks of acute respiratory viral diseases, such as influenza and COVID-19 caused by influenza A virus (IAV) and SARS-CoV-2, pose a serious threat to global public health, economic security, and social stability. This calls for the development of broad-spectrum antivirals to prevent or treat infection or co-infection of IAV and SARS-CoV-2. Hemagglutinin (HA) on IAV and spike (S) protein on SARS-CoV-2, which contain various types of glycans, play crucial roles in mediating viral entry into host cells. Therefore, they are key targets for the development of carbohydrate-binding protein-based antivirals. This study demonstrated that griffithsin (GRFT) and the GRFT-based bivalent entry inhibitor GL25E (GRFT-L25-EK1) showed broad-spectrum antiviral effects against IAV infection *in vitro* by binding to HA in a carbohydrate-dependent manner and effectively protected mice from lethal IAV infection. Although both GRFT and GL25E could inhibit infection of SARS-CoV-2 Omicron variants, GL25E proved to be significantly more effective than GRFT and EK1 alone. Furthermore, GL25E effectively inhibited *in vitro* co-infection of IAV and SARS-CoV-2 and demonstrated good druggability, including favorable safety and stability profiles. These findings suggest that GL25E is a promising candidate for further development as a broad-spectrum antiviral drug for the prevention and treatment of infection or co-infection from IAV and SARS-CoV-2.

**IMPORTANCE:**

Influenza and COVID-19 are highly contagious respiratory illnesses caused by the influenza A virus (IAV) and SARS-CoV-2, respectively. IAV and SARS-CoV-2 co-infection exacerbates damage to lung tissue and leads to more severe clinical symptoms, thus calling for the development of broad-spectrum antivirals for combating IAV and SARS-CoV-2 infection or co-infection. Here we found that griffithsin (GRFT), a carbohydrate-binding protein, and GL25E, a recombinant protein consisting of GRFT, a 25 amino acid linker, and EK1, a broad-spectrum coronavirus inhibitor, could effectively inhibit IAV and SARS-CoV-2 infection and co-infection by targeting glycans on HA of IAV and spike (S) protein of SARS-CoV-2. GL25E is more effective than GRFT because GL25E can also interact with the HR1 domain in SARS-CoV-2 S protein. Furthermore, GL25E possesses favorable safety and stability profiles, suggesting that it is a promising candidate for development as a drug to prevent and treat IAV and SARS-CoV-2 infection or co-infection.

## INTRODUCTION

During respiratory infections, multiple viruses, including influenza virus (IV) and coronavirus (CoV), often coexist and interact with each other to cause increased severity of infection (1, 2). Influenza is an acute respiratory disease caused by influenza A virus (IAV) and influenza B virus in humans. The predominant strains currently circulating globally are influenza A(H1N1)pdm09, influenza A(H3N2), and influenza B (B/Victoria lineage) (https://www.who.int/toolkits/flunet). The highly pathogenic avian influenza (HPAI) A(H7N9) caused a large-scale zoonotic epidemic in 2013 (3), whereas A(H5N1) has shown extensive transmission and mutation in birds and mammals since 2021, raising serious concern of potential cross-species transmission of HPAI H5 to humans, possibly resulting in a pandemic in the near future (4–9).

The global pandemic of COVID-19 caused by severe acute respiratory syndrome coronavirus 2 (SARS-CoV-2) infection has posed significant threats to public health and economic development worldwide. SARS-CoV-2 Omicron variants and subvariants are continuing to emerge, resulting in faster transmission and stronger immune escape abilities. These outcomes have given rise to increased breakthrough infection and re-infection among both vaccinated and non-vaccinated, but previously infected, individuals (10, 11). In the context of simultaneous influenza and COVID-19 outbreaks, the co-infection of IAV and SARS-CoV-2 results in even more severe lung tissue damage and more serious clinical symptoms (12–17). This dilemma calls for the development of broad-spectrum antivirals able to treat or prevent co-infection of IAV and SARS-CoV-2.

Viral entry is the initial step of viral infection of host cells. The key protein on the viral surface that mediates viral entry is an important target for the development of virus entry inhibitor-based antiviral drugs. Both hemagglutinin (HA) of IAV and spike (S) protein of SARS-CoV-2 belong to class I fusion proteins. HA of IAV consists of two subunits, HA1 that is responsible for viral binding to the sialic acid receptors on host cells and HA2 which mediates membrane fusion. Similarly, the S1 subunit of SARS-CoV-2 S protein contains a receptor-binding domain that recognizes and binds to the host receptor angiotensin-converting enzyme 2, while S2 subunit mediates membrane fusion (18). Therefore, both IAV HA and SARS-CoV-2 S protein are promising targets for the development of broad-spectrum viral entry inhibitor-based antiviral drugs.

A virus can utilize glycosylation to conceal important epitopes present on its envelope protein, allowing it to evade the immune system and enhance its survival and transmission (19, 20). Conversely, a high density of glycans can be targeted by carbohydrate-binding proteins (CBPs), such as griffithsin (GRFT), a lectin derived from the red alga *Griffithsia* sp (21). GRFT has been shown to have broad-spectrum inhibitory effect on infection of enveloped viruses, including human immunodeficiency virus 1 (HIV-1) (21–23), Hantaan virus (24), and acute respiratory syndrome coronavirus (SARS-CoV) (25). We have previously demonstrated that GRFT is highly effective in inhibiting SARS-CoV-2 infection by binding to the glycans displayed on its S protein (26). Based on this discovery, we then constructed a dual-target fusion inhibitor designated GL25E, also known as GRFT-L25-EK1 by linking GRFT and EK1, a pan-coronavirus fusion peptide that can bind to the HR1 of human coronaviruses (HCoVs) (27), with a 25-amino-acid linker (L25). We found that GL25E was more effective than GRFT alone against SARS-CoV-2 because GL25E can bind to the glycans mainly on S1 protein via its GRFT part and interact with HR1 domain in S2 protein through its EK1 part (27). Given that the S protein of SARS-CoV-2 and HA protein of IAV bear different types of glycans (28, 29), we hypothesized that both CBP-based antiviral proteins, GRFT and GL25E, may be capable of binding these viral proteins to block their entry into the host cells and, thus, inhibit their mono-infection or co-infection.

In this study, we demonstrated that both GRFT and GL25E efficiently and broadly inhibited infection of divergent IAVs by binding to the glycans on their HA, particularly HA1 protein, and found that GL25E was more effective than either GRFT or EK1 alone in inhibiting infection of SARS-CoV-2 Omicron variants and subvariants. Notably, GL25E effectively inhibited the *in vitro* co-infection of IAV and SARS-CoV-2 and demonstrated favorable druggability, including acceptable *in vitro* and *in vivo* safety and stability profiles. Therefore, GL25E has been demonstrated as a promising candidate for development as a safe and effective broad-spectrum antiviral for the prevention and treatment of IAV and SARS-CoV-2 mono- and co-infection.

## RESULTS

### Potent inhibitory activity of GRFT and GL25E against pseudotyped and authentic IAV infection *in vitro*

**Fig 1:**
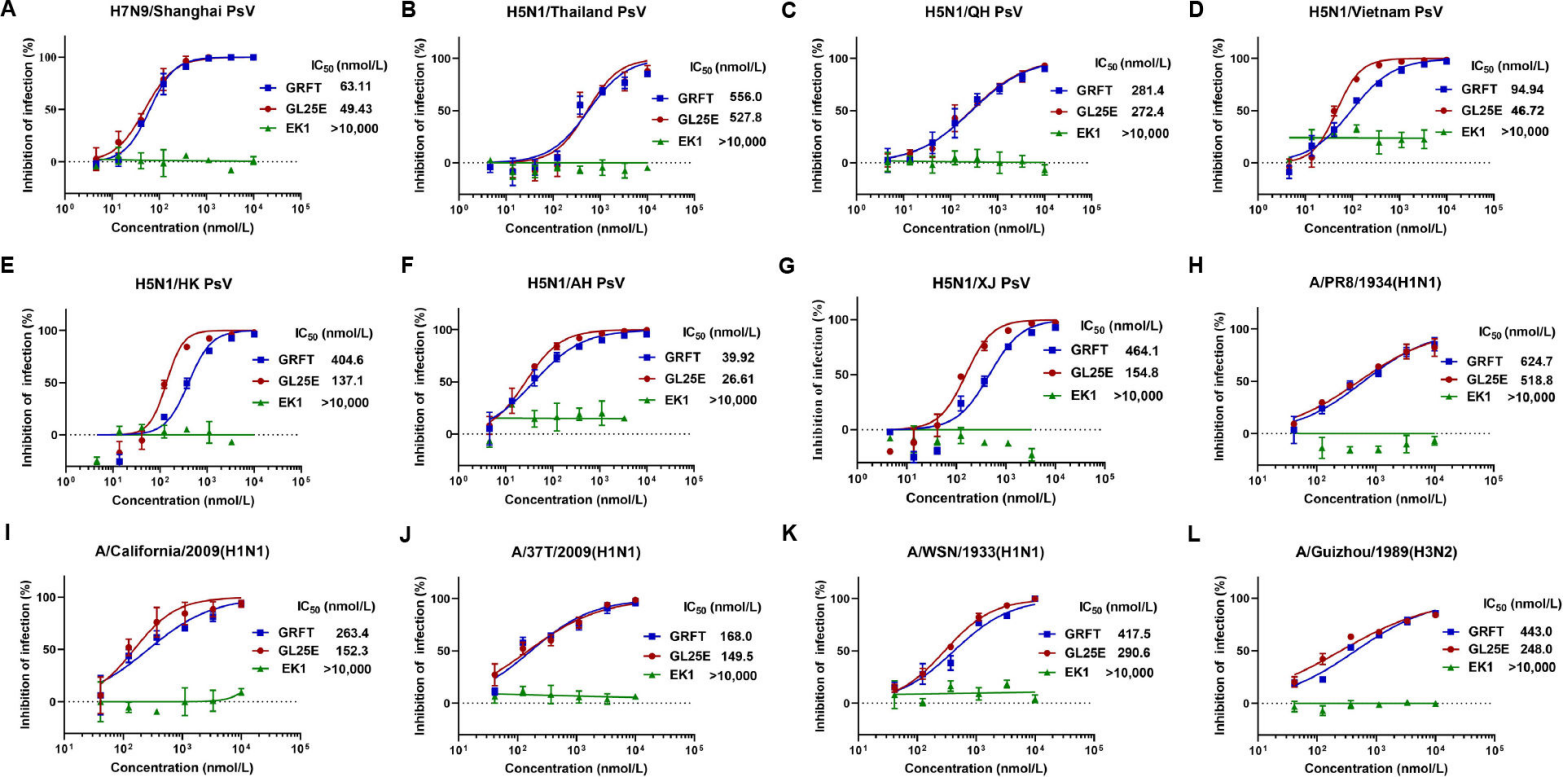
Inhibition of GRFT and GL25E on *in vitro* infection of divergent IAVs. (**A–G**) Inhibition of IAV H7N9 and H5N1 PsV infection was detected by luciferase assay. (**H–L**) Inhibition of infection of authentic IAVs, including A/Puerto Rico/8/1934(H1N1), A/California/04/2009(H1N1), A/Shanghai/37T/2009(H1N1), A/WSN/1933(H1N1), and A/Guizhou/54/1989(H3N2), was detected by plaque-reduction assay. Samples were tested in triplicate, and the experiment was repeated at least twice. Data from a representative experiment are expressed as means ± SD.

To evaluate the effectiveness of GRFT and GL25E in inhibiting IAV infection, we constructed a series of HIV-based pseudoviruses (PsVs) bearing the HA protein of H5 and H7 subtypes of IAVs, including H5N1/Thailand, H5N1/QH, H5N1/Vietnam, H5N1/HK, H5N1/AH, H5N1/XJ, and H7N9/Shanghai. We found that both GRFT and GL25E exhibited potent inhibitory activity against infection of the above IAV PsVs with half maximal inhibitory concentration (IC_50_) ranging from 39.9 to 556 nmol/liter and from 26.6 to 528 nmol/liter, respectively (Fig. 1A through G).

We then assessed the inhibitory activity of GRFT and GL25E on infection of authentic IAV, including H1 and H3 subtypes, such as A/Puerto Rico/8/1934(H1N1), A/California/07/2009(H1N1), A/Shanghai/37T/2009(H1N1), A/WSN/1933(H1N1), and A/Guizhou/54/1989(H3N2). As shown in Fig. 1H through L, both GRFT and GL25E were effective in inhibiting authentic IAV infection in a dose-dependent manner with the IC_50_ ranging from 168 to 625 nmol/liter and from 150 to 519 nmol/liter, respectively. However, EK1 alone exhibited no inhibitory activity against infection of either pseudotyped or authentic IAVs at the concentration up to 10,000 nmol/liter. Further investigation revealed that both GRFT and GL25E effectively inhibited authentic IAV infection as determined by indirect immunofluorescence assay (Fig. S1) and Westen blot assay on MDCK, A549, and Calu-3 cells (Fig. S2). These findings indicate that both GRFT and GL25E exhibit inhibitory activity against IAV infection *in vitro*, possibly by targeting the HA of two major phylogenetic subgroups: group 1 (H1 and H5) and group 2 (H3 and H7).

### Prophylactic or therapeutic effect of GRFT and GL25E on the protection of mice against challenge with IAV A/PR8/H1N1

**Fig 2:**
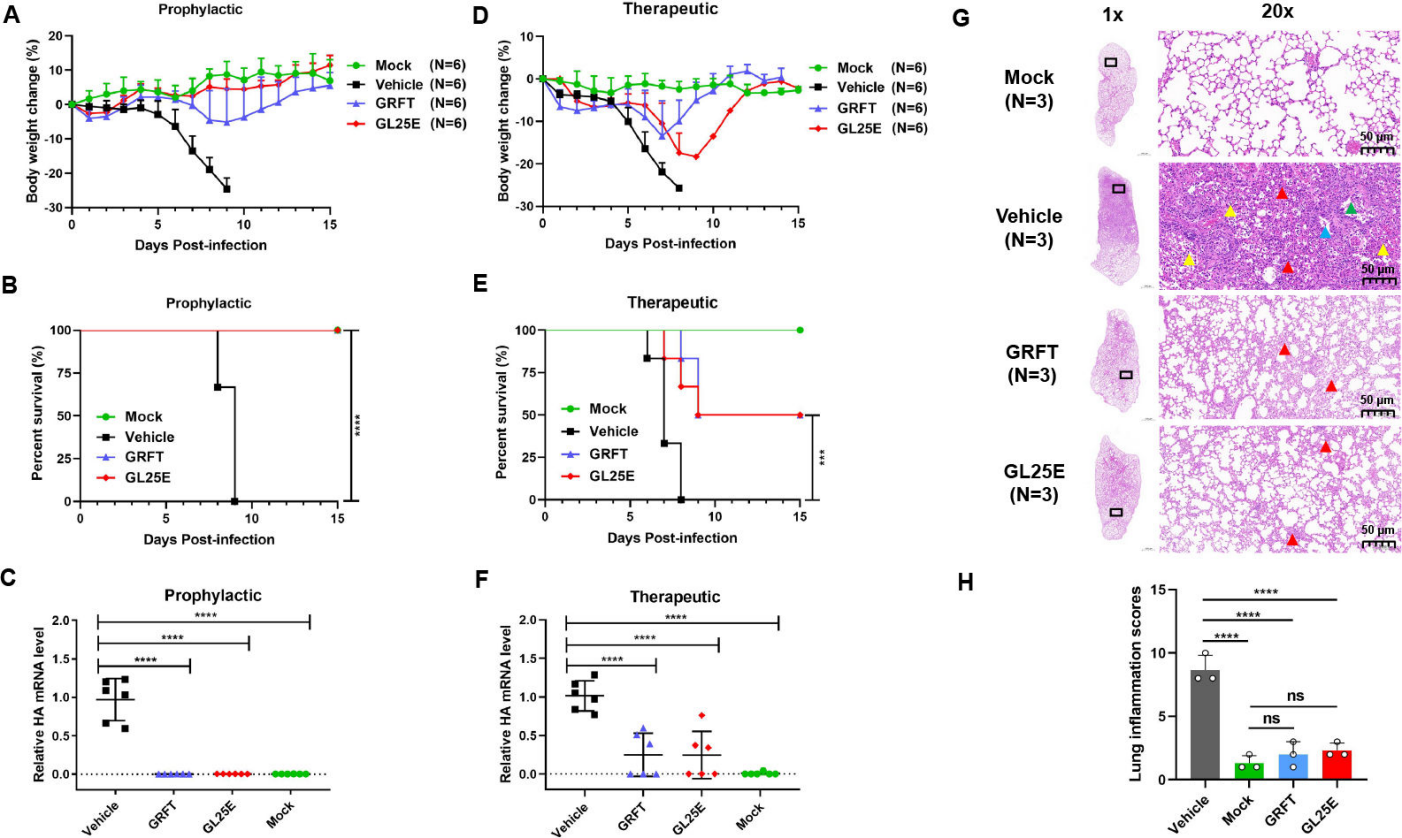
Prophylactic and therapeutic effect of GRFT or GL25E against A/Puerto Rico/8/1934 (H1N1) infection in C57BL/6J mice. Female C57BL/6J mice were intranasally administered with 10 mg/kg of GRFT or GL25E 30 min before or after intranasal challenge with 176 PFU of A/Puerto Rico/8/1934 (H1N1). Each group had 15 mice, including 6 mice for detection of body weight change and survival rate, 6 mice for evaluation of viral load, and 3 mice for histological study. (A–C) Body weight change (%) (**A**), survival rate (%) (**B**), and viral load (**C**) in mice of the prophylactic treatment group. (D–F) Body weight change (%) (**D**), survival rate (%) (**E**), and viral load (**F**) in mice of the therapeutic treatment group. Data are presented as means ± SD. (**G**) Representative photomicrographs of lung tissue in prophylactic group (hematoxylin and eosin, H&E staining). Scale bars = 1,000 µm (1×, left) and scale bar  = 50 µm (20×, right). (**H**) Histologic scores. Overall histologic score is calculated by adding up the scores for each individual criterion (hemorrhage, neutrophil infiltration, thickness of the alveolar wall, and atelectasis). Each sample was tested in triplicate, and the experiment was repeated at least twice. Data from a representative experiment are presented as means ± SD. A significant difference between groups was analyzed using one-way ANOVA. **P < 0.01; ***P < 0.001; ****P < 0.0001.

We then determined whether GRFT and GL25E were effective in protecting mice from infection of IAV A/PR8/H1N1, a mouse-adapted strain of A/Puerto Rico/8/1934(H1N1). GRFT and GL25E as prophylactic or therapeutic agents diluted in PBS were intranasally administered to C57BL/6J mice at a dose of 10 mg/kg, either 0.5 h before or after IAV challenge for prevention or treatment, respectively, while mice receiving PBS only before or after IAV challenge and those receiving PBS only without IAV challenge were tested as vehicle control group and mock infection group, respectively. In the prophylactic experiment, all mice in the vehicle control group died on day 9 after infection and showed continuous weight loss starting from day 5 post-infection. However, pretreatment with GRFT or GL25E delayed, or reversed, body weight loss and effectively protected the mice from death (Fig. 2A and B). In the therapeutic experiment, mice administered with GRFT or GL25E exhibited a 50% survival rate with a reduced body weight loss, while no mice in the vehicle control group survived by day 8 post-infection (Fig. 2D and E). To further confirm the protective effect, we also assessed viral load in the lungs using real-time PCR. As depicted in Fig. 2C and F, treatment with GRFT or GL25E significantly reduced viral load in the lung, and prophylactic effect was superior to the therapeutic effect. Additionally, mice treated with GRFT or GL25E exhibited only minor lung damage compared with mice in the vehicle control group in prophylactic group (Fig. 2G). No histopathological changes were observed in the mock-infected group, while mice in the vehicle control group displayed significantly increased histopathological changes in the lung with indistinct outlines of large areas of alveoli in the field of vision and structural disorder (red arrow). Some epithelial cells were detached from the bronchioles (blue arrow), and a few erythrocytes and foam cells were visible in the lumen (green arrow). A significant number of inflammatory cells infiltrated the tissue (yellow arrow). In contrast, mice in the GRFT and GL25E treatment groups showed mildly abnormal structure with no apparent infiltration of inflammatory cells and slight thickening of the alveolar wall in the field of vision. Pathologists performed a blinded qualitative histological assessment of lung tissues, and the mean histological scores are presented in Fig. 2H. The GRFT- and GL25E-treatment groups had significantly lower scores compared with the vehicle control group (Table S1). These results indicate that both GRFT and GL25E possess inhibitory activity against IAV infection *in vivo*.

### Inhibition of IAV infection by GRFT and GL25E through interfering with the viral entry stage

**Fig 3:**
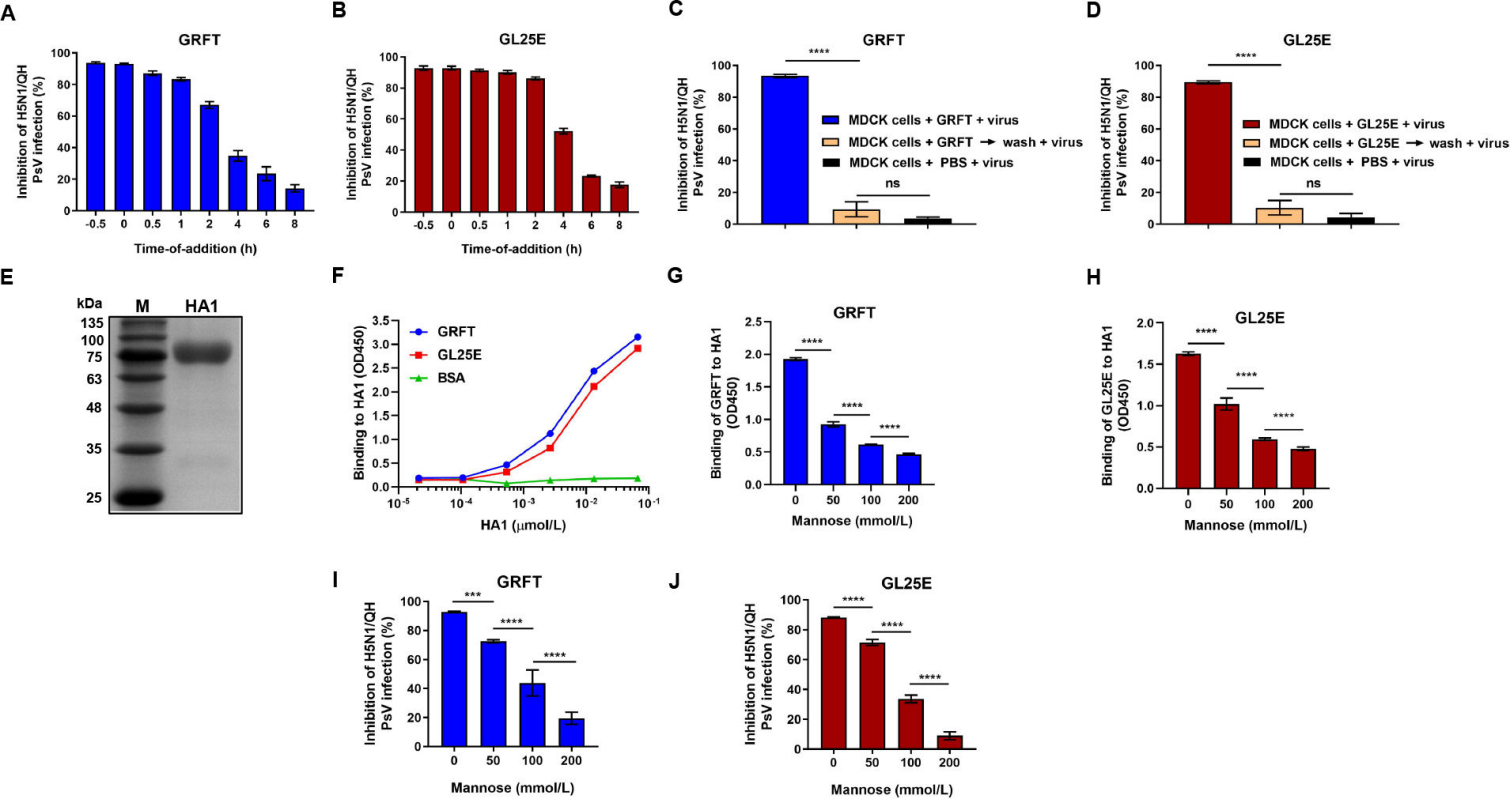
Mechanism of action of GL25E in inhibiting IAV infection. (**A and B**) Time-of-addition assay: MDCK cells were treated with GRFT (**A**) or GL25E (**B**) at the indicated time points before or after the addition of pseudotyped H5N1/QH. (**C and D**) Washout assay: MDCK cells were pretreated with 10 µmol/liter of GRFT or GL25E at 37° for 1 h before cells were washed with PBS to remove unbound inhibitor. Subsequently, cells were infected with pseudotyped IAV H5N1/QH. In the other group, inhibitor-pretreated or PBS-treated cells were not washed before the addition of IAV H5N1/QH PsV. The inhibitory activity of GRFT and GL25E on IAV PsV infection was assessed using a luciferase assay. (**E**) Analysis of expressed recombinant HA1 protein using SDS-PAGE. (**F**) Measurement of the binding of HA1 protein to GRFT or GL25E using enzyme-linked immunosorbent assay (ELISA). (**G and H**) Measurement of the effect of mannose on the binding of GRFT or GL25E to HA1 using ELISA. (**I and J**) Measurement of the effect of mannose on GRFT- or GL25E-mediated inhibition of H5N1/QH PsV infection using a luciferase assay. Each sample was tested in triplicate, and the experiment was repeated at least twice. Data from a representative experiment are presented as means ± SD. Statistical analysis was performed and analyzed using one-way ANOVA. *P < 0.05; **P < 0.01; ***P < 0.001; ****P < 0.0001; ns, not significant.

A time-of-addition assay was conducted to determine which stage in the IAV life cycle GRFT and GL25E may target. As shown in Fig. 3A and B, GRFT and GL25E exhibited over 70% inhibition of H5N1/QH PsV infection when the inhibitor was added to cells at −0.5, 0, 0.5, 1 and 2 h after IAV PsV infection, while their inhibitory activity decreased quickly at 4 and 6 h post-IAV PsV infection, suggesting that GRFT and GL25E inhibit IAV infection at the viral entry stage. To further elucidate whether GRFT and GL25E act on virus or cells, a washout assay was performed. As shown in Fig. 3C and D, no inhibition of H5N1/QH PsV infection was observed when the virus was added to cells after inhibitor-pretreated cells were washed, indicating that GRFT and GL25E inhibit virus infection by acting on the virus, not the host cells.

We then investigated the potential interaction between these inhibitors and HA1, considering that hemagglutinin 1 (HA1) plays an important role in IAV entry into host cells. Recombinant HA1 protein was expressed and purified, as previously described (30), and then identified using SDS-PAGE (Fig. 3E). An enzyme-linked immunosorbent assay (ELISA) was performed to assess the binding ability of GRFT or GL25E to HA1. As shown in Fig. 3F, GRFT or GL25E bound to HA1 in a dose-dependent manner, confirming that HA1 is a target for these inhibitors.

We have previously demonstrated that GRFT and GL25E inhibit SARS-CoV-2 infection by binding the glycans in its S1 protein, demonstrating that mannose can attenuate antiviral activity of the glycan-binding proteins GRFT and GL25E (26, 27). In this study, we evaluated whether mannose could interfere with the interaction between these inhibitors and HA1. As shown in Fig. 3G and H, mannose did exhibit dose-dependent reduction in the binding of GRFT or GL25E to HA1. Similarly, mannose attenuated the inhibitory effect of GRFT and GL25E on IAV PsV infection in a dose-dependent manner (Fig. 3I and J). These findings suggest that GRFT and GL25E inhibit IAV infection by binding to the glycans presented on HA1.

### Efficacy of GL25E over that of GRFT and EK1 alone in inhibiting the infection of SARS-CoV-2 Omicron variants and subvariants

**Fig 4:**
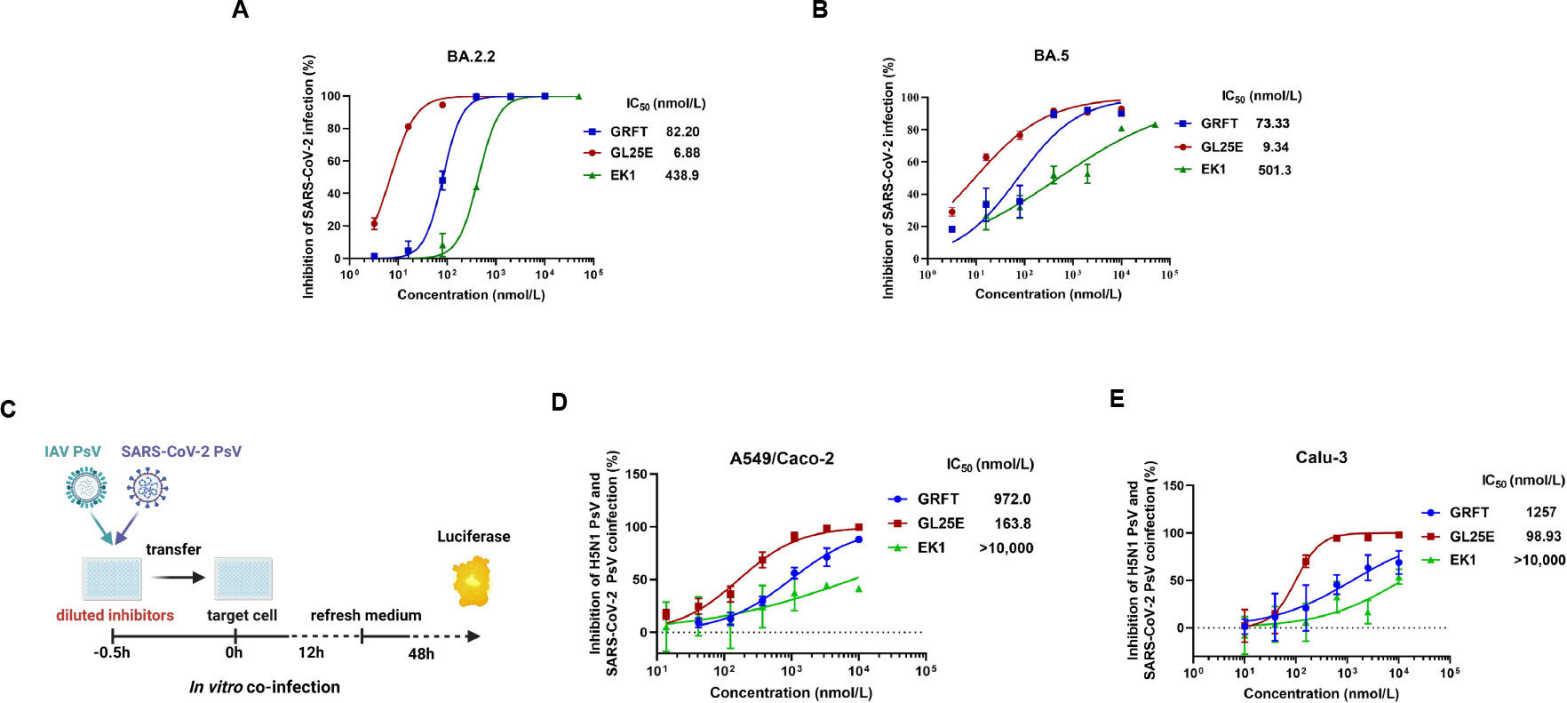
Inhibition of GRFT or GL25E on the co-infection of IAV and SARS-CoV-2. (**A and B**) Inhibition of GRFT or GL25E (EK1 as a control) against the infection of authentic SARS-CoV-2 Omicron variants BA.2.2 (**A**) and BA.5 (**B**) in Caco-2 cells using an RT-qPCR assay for N genes. (**C**) Diagram illustrating the procedure for co-infection. (**D and E**) Inhibition of IAV and SARS-CoV-2 co-infection in the mixture of A549 and Caco-2 cells (**D**) or in Calu-3 cells (**E**). A mixture of pseudotyped IAV H5N1/Thailand and SARS-CoV-2 D614G at 1:1 ratio was added to cells in the presence of serially diluted GRFT, GL25E, or EK1. After 12 h, the culture medium was replaced, and the cells were incubated for an additional 48 h. Luciferase assay was performed to evaluate the inhibitory activity of GRFT, GL25E, or EK1 against PsV infection. Each sample was tested in triplicate, and the experiment was repeated at least twice. Data from a representative experiment are presented as means ± SD.

Our previous reports have shown that both GRFT and GL25E are effective in inhibiting infection of pseudotyped SARS-CoV-2 original strain and other HCoVs, including SARS-CoV, MERS-CoV, HCoV-OC43, HCoV-229E, and HCoV-NL63 (26, 27). Here, we tested the efficacy of GRFT, GL25E, and EK1 in inhibiting infection by SARS-CoV-2 Omicron variants (e.g., B.1.1.529, BA.2, BA.3, and BA.5) and subvariants (e.g., BA.2.2, BA.2.12.1, BA.2.9, BF.7, BQ.1.1, and XBB). As shown in Table 1, GL25E exhibited potent inhibitory activity against infection of SARS-CoV-2 Omicron variants and subvariants with an IC_50_ ranging from 20 to 58 nmol/liter, while GRFT or EK1 alone showed less inhibitory activity with IC_50_ values in the ranges of 90–2,920 and 37–350 nmol/liter, respectively. Furthermore, GL25E was more effective than either GRFT or EK1 against infection by authentic SARS-CoV-2 Omicron subvariant BA.2.2 with IC_50_ values of 6.88, 82.2, and 439 nmol/liter, respectively (Fig. 4A), and subvariant BA.5 with IC_50_ values of 9.34, 73.3, and 501 nmol/liter, respectively (Fig. 4B). In the plaque-reduction assay (PRA), GL25E seemed more potent than GRFT against infection of authentic SARS-CoV-2 Omicron variant BA.5 and subvariant BA.2.2 although there is no statistically significant difference (Table S2).

**TABLE 1:** Inhibitory activity of GRFT, GL25E, and EK1 against infection of SARS-CoV-2 Omicron variant PsV^*a*^

Pseudovirus	IC_50_ (nmol/liter)		
	GRFT	GL25E	EK1
B.1.1.529	1,378	41.68	200.4
BA.2	270.4	29.16	125.9
BA.2.2	772.7	32.49	259.3
BA.2.12.1	117.2	26.59	307.3
BA.2.9.1	1,184	26.96	350.4
BA.3	2,902	58.35	270.6
BA.5	256.9	56.78	231.6
BF.7	89.69	24.19	36.69
BQ.1.1	712.5	56.20	72.31
XBB	293.6	20.33	37.82

^*a*^ IC_50_: half maximal inhibitory concentration.

### Efficacy of GL25E over that of GRFT in inhibiting IAV and SARS-CoV-2 co-infection

To determine whether GL25E is effective in inhibiting IAV and SARS-CoV-2 co-infection, we performed *in vitro* co-infection models (Fig. 4C). Briefly, GL25E at graded concentration was incubated with both pseudotyped IAV and SARS-CoV-2 before the addition of the inhibitor/virus mixture to cultured A549 cells susceptible to IAV and Caco-2 cells susceptible to SARS-CoV-2, or to cultured Calu-3 cells susceptible to both IAV and SARS-CoV-2. As shown in Fig. 4D, GL25E effectively inhibited the co-infection of pseudotyped IAV and SARS-CoV-2 in A549/Caco-2 cells with the IC_50_ of 164 nmol/liter, while GRFT was less effective against IAV and SARS-CoV-2 in A549/Caco-2 cells with IC_50_ of 972 nmol/liter. EK1 peptide exhibited less than 50% inhibition at the concentration up to 10,000 nmol/liter. Similarly, GL25E showed significant inhibition of IAV and SARS-CoV-2 co-infection in Calu-3 cells with IC_50_ of 194 nmol/liter, whereas GRFT was much less potent with IC_50_ of 1,257 nmol/liter. Again, EK1 showed no significant inhibition at the concentration up to 10,000 nmol/liter (Fig. 4E).

### Good safety and stability profile of GL25E

**Fig 5:**
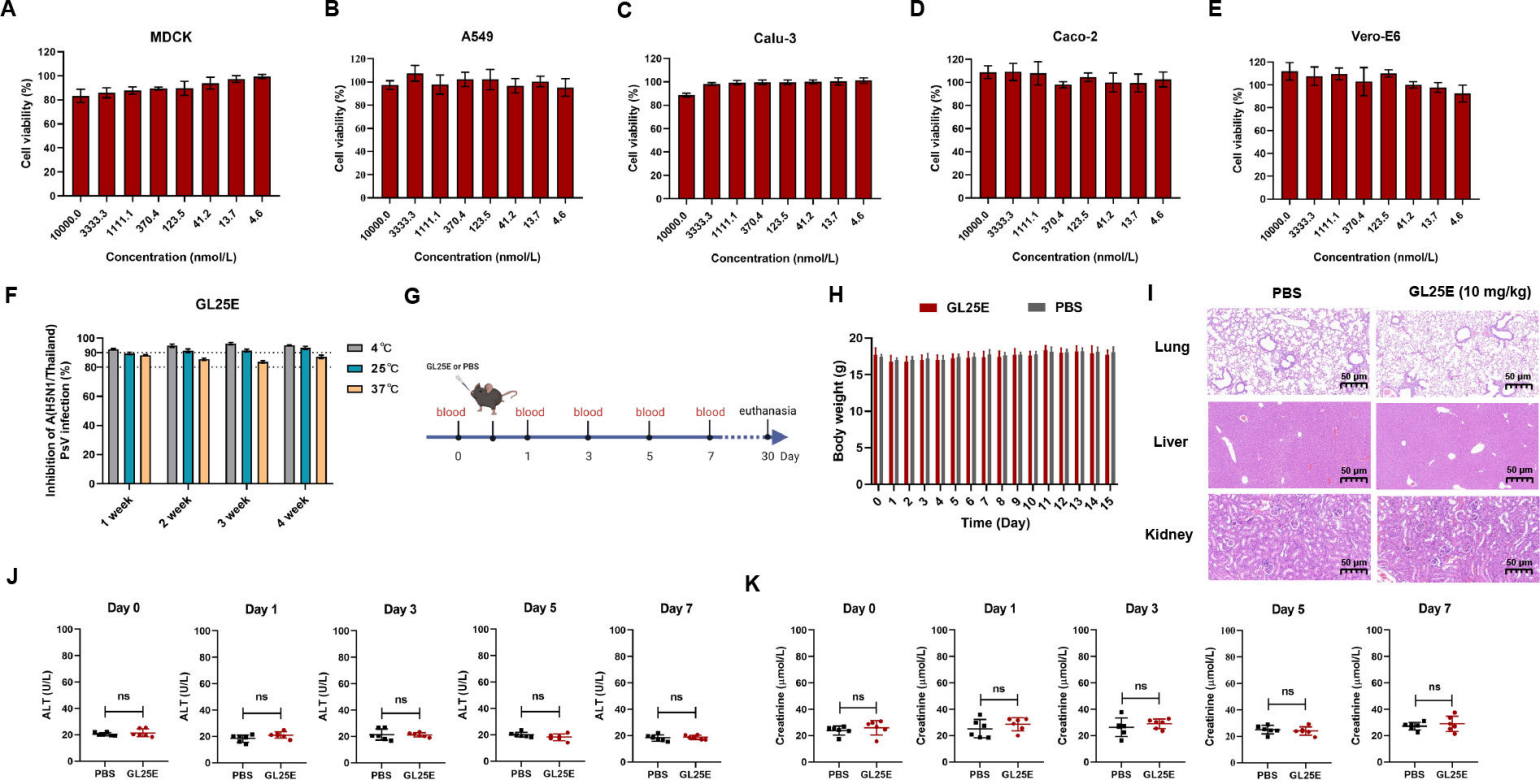
*In vitro* and *in vivo* safety and thermal stability of GL25E. (**A–E**) Cytotoxicity of GL25E on MDCK, A549, Calu-3, Caco-2, and Vero-E6 cells, respectively. (**F**) Stability of GL25E stored at 4, 25, and 37°C for 1, 2, 3, and 4 weeks, respectively, as determined by testing their its inhibitory activity against A/H5N1/Thailand PsV infection. (**G**) Diagram of the *in vivo* safety experiment procedure. (**H**) Monitoring of murine body weight changes once daily for 15 days after intranasal administration of GL25E (10 mg/kg). (**I**) Histopathological changes in murine lungs, liver, and kidneys were observed through H&E staining after intranasal administration of GL25E (10 mg/kg) or PBS (as a control) on day 30 in mice (*n* = 3). (**J and K**) The levels of ALT (U/L) (J) and creatinine (K) in sera collected from mice on days 0, 1, 3, 5, and 7, respectively. Each sample was tested in triplicate, and the experiment was repeated at least twice. Data from a representative experiment are presented as means ± SD. “ns” denotes no significance.

Finally, we evaluated the *in vitro* and *in vivo* safety and stability of GL25E, as previously described (31). As shown in Fig. 5A through E, the viability of MDCK, A549, Calcu-3, Caco-2, and Vero-E6 cells remained above 80% after 48 h of treatment with GL25E at concentrations up to 10 µmol/liter. We then investigated the *in vitro* stability of GL25E by testing its inhibitory activity against IAV PsV infection after storage at 4, 25, and 37℃ for different durations. As shown in Fig. 5F, GL25E retained remarkable antiviral activity after storage at 4, 25, and 37°C for 1–4 weeks, respectively, confirming its high *in vitro* stability.

Subsequently, we assessed *in vivo* safety in C57BL/6J mice receiving intranasal administration of GL25E in PBS (10 mg/kg) or PBS as control, as previously described (32). Body weight changes were monitored for the next 2 weeks, and serum samples were collected on days 0, 1, 3, 5, and 7 to detect the levels of alanine aminotransferase (ALT) and creatinine. On day 30, mice were euthanized for collection of lungs, livers, and kidneys for hematoxylin and eosin (H&E) staining (Fig. 5G). As shown in Fig. 5H through K, no significant differences were observed in body weight loss, histopathology, or serum levels of ALT and creatinine between mice administered with GL25E in PBS and PBS control, indicating that GL25E also possesses a satisfactory *in vivo* safety profile.

To detect the *in vivo* distribution and stability of GL25E, we intranasally administered 10 mg/kg of Cy5-GL25E or PBS as a control to C57BL/6J mice and monitored the distribution of Cy5-GL25E in the lung, kidney, heart, spleen, and liver at 3, 24, 48, 72, and 96 h, respectively, using the IVIS Lumina K Series III *in vivo* imaging system. As shown in Fig. S3A, the fluorescence signals of Cy5-GL25E were mainly observed in the lung after intranasal administration of GL25E up to 96 h post-treatment but not in other organs. After mice were dissected at 48 h after administration, the fluorescence in various organs was measured. As shown in Fig. S3B and S3C, the Cy5-GL25E fluorescence signals were predominantly concentrated in the lungs, while those in other organs were minimal, similar to those in the PBS control group. These results suggest that intranasally administered GL25E is mainly distributed in lung for up to 96 h.

## DISCUSSION

Measures implemented during the COVID-19 pandemic, such as lockdowns and facemask mandates, may make individuals gain “immunity debt,” thus becoming more susceptible to influenza virus infections (33, 34). For example, a significant wave of influenza virus infections with a peak infection rate of 37.3% in 2023 was reported in China (35). Simultaneously, SARS-CoV-2 Omicron subvariants have continuously emerged with high immune evasion capacity and transmissibility, such as XBB.1.5, BA.2.86, and JN.1 (11, 36). Even worse, prior infection with IAV can significantly exacerbate the infectivity and pathogenesis of SARS-CoV-2 in co-infected patients (14, 15, 37). These recent discoveries have intensified the need to develop effective and broad-spectrum antiviral agents to combat mono- and co-infection of influenza virus and SARS-CoV-2.

Currently, numerous antiviral drugs have been approved for the treatment or prevention of SARS-CoV-2 infection, including two classes of viral replication inhibitors, main protease (M^pro^) and RNA-dependent RNA polymerase inhibitors, and one class of viral entry inhibitors, S protein-specific neutralizing antibodies (nAbs) (38). Because of their extensive S protein mutations, Omicron variants and subvariants have become resistant to most nAbs under emergency use authorization (39). Besides, these anti-SARS-CoV-2 nAbs are ineffective against IAV infection. Therefore, it is essential to develop broad-spectrum viral entry inhibitors with activity against co-infection of divergent IAV and SARS-CoV-2 strains.

In this study, we demonstrated that both GRFT and the GRFT - based bivalent fusion inhibitor GL25E could effectively inhibit *in vitro* infection of divergent pseudotyped and authentic IAVs, as well as show prophylactic and therapeutic effect against *in vivo* infection of the authentic A/PR8/H1N1 in C57BL/6J mice. While both GRFT and GL25E were effective, GL25E was significantly more potent than GRFT in inhibiting infection of such pseudotyped SARS-CoV-2 Omicron variants as B.1.1.529, BA.2, BA.3, and BA.5 and subvariants, including BA.2.2, BA.2.12.1, BA.2.9, BF.7, BQ.1.1, and XBB, which are resistant to most nAbs under emergency use authorization, as well as antibodies in sera from SARS-CoV-2-infected or -vaccinated individuals (10, 11, 39). GL25E was also more efficient than GRFT against IAV and SARS-CoV-2 co-infection *in vitro*.

**Fig 6:**
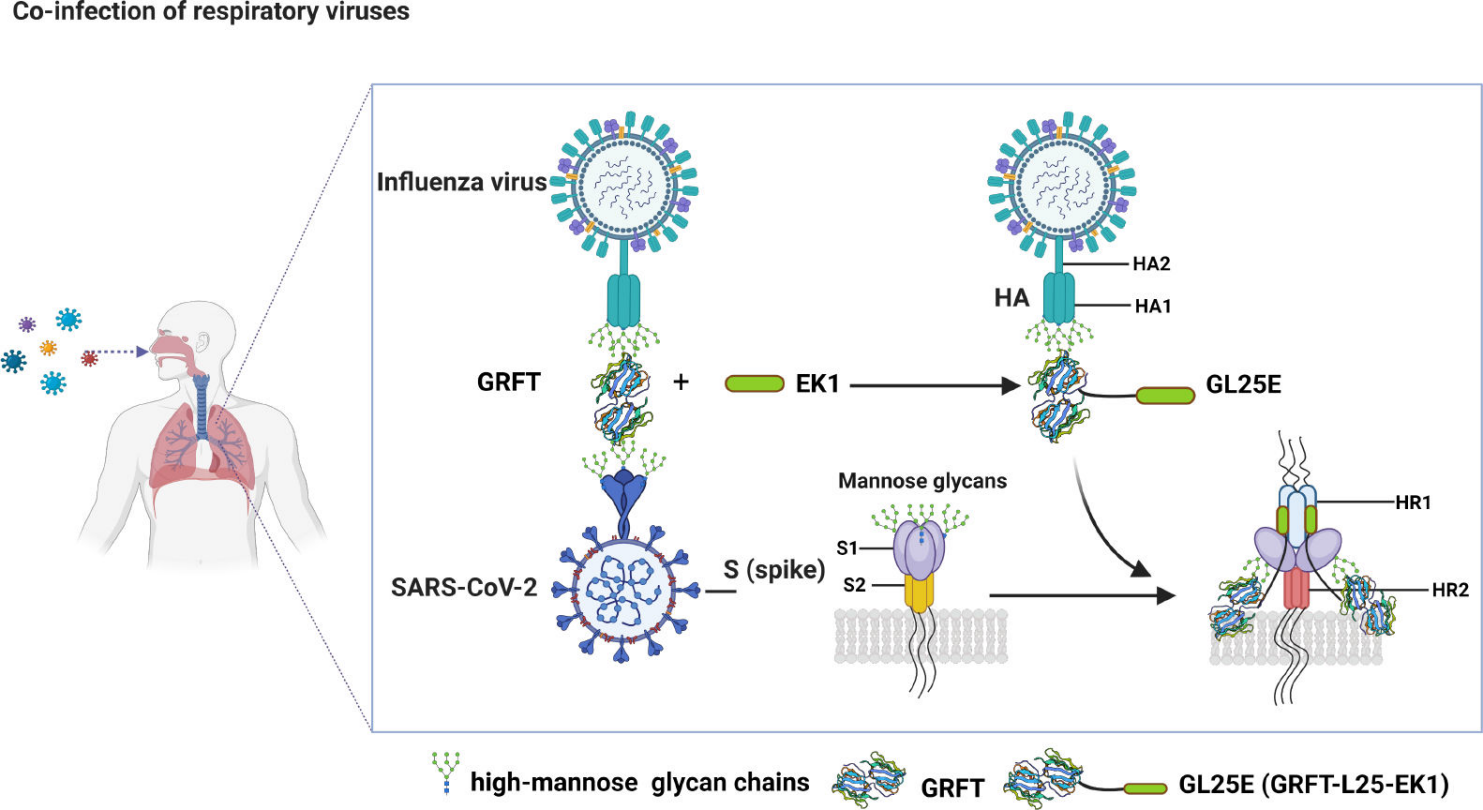
Schematic illustration of the mechanism by which GRFT and GL25E inhibit mono- or co-infection of IAV and SARS-CoV-2. GRFT or GRFT part in GL25E binds to the glycans on HA1 of IAV, thereby inhibiting IAV entry into the host cell. GL25E inhibits SARS-CoV-2 infection by binding via its GRFT part to glycans on S1 subunit of SARS-CoV-2 S protein and interacting via its EK1 part with HR1 domain in S2 subunit of SARS-CoV-2 S protein. GRFT (PDB code: 7RID).

We observed that the presence of mannose attenuated inhibitory activity against IAV infection, suggesting that the glycans displayed on HA1 were the target of GRFT. These findings highlight the potential of GRFT and GL25E as therapeutics and prophylactics for controlling influenza and COVID-19 pandemics. Interestingly, GL25E exhibited stronger inhibitory activity against SARS-CoV-2 variants in a carbohydrate-dependent manner. This confirms our hypothesis that the glycans displayed on S protein and HA could be promising targets for the development of antiviral drugs (Fig. 6). Antiviral strategies commonly involve the utilization of vaccines and antiviral medications. Influenza virus vaccines can be effective in reducing morbidity and mortality, but their clinical use is limited because of the high variability of influenza viruses. For antiviral drugs, the Centers for Disease Control and Prevention recommends Oseltamivir phosphate, Zanamivir, Peramivir, and Baloxavir marboxil to treat influenza virus infection. However, the emergence of drug-resistant influenza strains can render these drugs ineffective (40, 41).

Mechanistic studies have shown that GRFT and GL25E inhibit IAV and SARS-CoV-2 infection by blocking viral entry into the host cells in a mannose-dependent manner, suggesting that both GRFT and GL25E bind to and cross-link glycans in the HA of IAV, particularly HA1, and S protein of SARS-CoV-2, especially S1 subunit, on the surface of viral particles, preventing them from undergoing structural changes or accessing the cell surface receptor. The effectiveness of this approach is supported by the fact that a glycan-targeted nAb 2G12 has been shown to inhibit both HIV and IAV infection (42). GL25E is significantly more effective than GRFT in inhibiting the mono- and co-infection of IAV and SARS-CoV-2 because GL25E can target the glycans mainly in HA1 of IAV, S1 subunit of SARS-CoV-2, and HR1 domain in S2 subunit of SARS-CoV-2. Its inhibitory potency is generally unaffected by protein sequence variations in divergent IAV and SARS-CoV-2 strains. This unique characteristic of GL25E enhances its broad-spectrum activity against IAV and SARS-CoV-2 infection. Additionally, GL25E is cost-effective because it can be produced at a large scale using an *Escherichia coli* expression system. Moreover, GL25E exhibits excellent *in vitro* and *in vivo* safety and stability, along with good druggability. Therefore, GL25E is a promising candidate for further development as a broad-spectrum antiviral drug for the treatment and prevention of mono- or co-infection of IAV and SARS-CoV-2.

## MATERIALS AND METHODS

### Cells, viruses, and peptides

MDCK, 293T, A549, Calu-3, Vero-E6, and Caco-2 cells were obtained from ATCC and cultured with DMEM containing 10% fetal bovine serum. The influenza virus strains A/Puerto Rico/8/1934(H1N1), A/Guizhou/54/1989(H3N2), A/California/07/2009(H1N1), A/Shanghai/37T/2009(H1N1), and A/WSN/1933(H1N1) were acquired from BEI Resources. Live influenza viruses were propagated in MDCK cells, and virus titers were determined using a plaque formation assay. Authentic SARS-CoV-2 Omicron variants BA.2.2 and BA.5 were maintained in the BSL-3 Laboratory of Shanghai Medical College, Fudan University. The EK1 peptide used in this study was synthesized by Chengdu Shengnuo Biotechnology Co., Ltd. (Chengdu, China), and its sequence was previously described (31).

### Plasmids

The plasmids pVKD-HA and pVKD-NA used for encoding the envelope proteins of IAV strain A/Shanghai/4664T/2013(H7N9) were obtained from the Shanghai Public Health Clinical Center. Plasmids encoding the HA proteins of various H5N1 strains, including A/QH/59/05, A/Thailand/Kan353/2004, A/Anhui/1/2005, A/Hong Kong/156/97, A/Vietnam/1194/2004, and A/Xinjiang/1/2006, as well as the N1-typed NA protein of A/Thailand/Kan353/2004. All these plasmids were constructed as previously described (43–45). The following plasmids encoding S protein of coronaviruses, including pcDNA3.1-SARS-CoV-2-S, pcDNA3.1-SARS-CoV-2-S D614G, pcDNA3.1-B.1.1.529-S, pcDNA3.1-BA.2-S, pcDNA3.1-BA.2.12.1-S, pcDNA3.1-BA.2.2-S, pcDNA3.1-BA.2.9.1-S, pcDNA3.1-BA.3-S, pcDNA3.1-BA.4/5-S, pcDNA3.1-BF.7-S, pcDNA3.1-BQ.1.1-S, and pcDNA3.1-XBB-S. The plasmid pNL4-3.Luc.R^-^.E^-^ was used as HIV-backbone-based luciferase reporter vector.

### Production of pseudoviruses

IAV or SARS-CoV-2 PsVs were generated as previously described (45, 46). Briefly, HEK293T cells were co-transfected with pNL4-3.luc.R^-^E^-^ and plasmids encoding the HA and NA proteins of IAV, or pcDNA3.1-plasmids encoding S protein of various SARS-CoV-2 strains, respectively. Supernatants containing PsVs were collected and centrifuged at 3,000 × *g* for 10 min.

### Plaque reduction assay

Antiviral activity of GRFT and GL25E on IAV infection was measured using a PRA (47). Briefly, IAVs were treated with GRFT, GL25E, or EK1 peptide at the indicated concentration for 1 h at 37°C, and inhibitor-virus mixture was transferred to MDCK cells. At 2 h post-infection, supernatants were removed and covered with DMEM containing 1% low melting point agarose (Invitrogen, Carlsbad, CA, USA) and 1 µg/mL TPCK-trypsin. After 2–3 days, the cell monolayer was fixed and stained with 4% paraformaldehyde containing 0.5% crystal violet for 4 h, and the number of plaques was counted.

### Inhibition of IAV PsV or SARS-CoV-2 PsV infection

The inhibition of infection by IAV PsVs or SARS-CoV-2 PsVs was performed as described previously (48, 49). In brief, pseudotyped IAV or SARS-CoV-2 Omicron variant was mixed with an equal volume of the tested proteins or peptide at the indicated concentration and incubated for 30 min before being transferred to the target cells (MDCK cells for IAV PsVs and Caco-2 cells for SARS-CoV-2) for 12 h. Supernatants were removed, and target cells were cultured with fresh DMEM medium for 48 h. Target cells were lysed, and inhibitory activity was determined using a Luciferase Assay System (Promega, Madison, WI, USA).

### Enzyme-linked immunosorbent assay

The interaction between HA1 protein and GRFT or GL25E was evaluated using ELISA as previously described (50). Plates were coated with GRFT, GL25E, and BSA in PBS and incubated at 4°C overnight and blocked with 2% gelatin at 37°C for 2 h. HA1 was added and incubated at 37°C for 1 h. Detection was done using HRP-conjugated goat anti-human IgG at 37°C for 1 h. Inhibition of GRFT/GL25E binding to HA1 by mannose was assessed by pre-treating GRFT or GL25E with mannose at indicated concentrations and then adding the mixture to plates coated with HA1 for 1 h. Detection was performed using HRP-conjugated anti-6 His antibody at 37°C for 1 h (27).

### *In vivo* inhibition of authentic IAV infection

Female C57BL/6J mice (6–8 weeks old) were purchased from Charles River (Beijing, China) and divided into four groups (15 mice/group): mock-infection, vehicle, GRFT, and GL25E. Mice in the mock-infection group were not exposed to IAV, while those in the other groups were challenged with IAV as previously described (51). To evaluate the prophylactic or therapeutic effects of GRFT and GL25E, intranasal administration of GRFT or GL25E at 10 mg/kg, or PBS (vehicle) was performed before or after challenge with A/Puerto Rico/8/1934 (4.4 × 10^3^ PFU/mL). Changes in body weight and survival rate of the mice were recorded daily up to 15 days after infection (*n* = 6). Three mice from each group were euthanized 7 days post-infection, and the lungs were removed and fixed in 4% paraformaldehyde at 4℃ for H&E staining and histological analysis based on the following indicators: alveolar edema, hemorrhage, neutrophil infiltration, hyaline membrane formation, thickness of the alveolar wall, and atelectasis. The scoring system assessed the level of damage on a scale ranging from 0 (not damage observed) to 3 (severe damage). Six mice from each group were euthanized for examination of viral titer in the lungs 7 days post-infection. Total RNA was extracted from the lung homogenates, and the levels of viral RNA were determined using a One-Step qRT-PCR kit (Takara Bio, Shiga, Japan) with primer sequences listed in Table S3.

### Time-of-addition assay and washout assay

Time-of-addition assay was performed as described previously (52). Briefly, MDCK cells were seeded into wells of a 96-well plate. GRFT or GL25E was added at a final concentration of 10 µmol/liter either 0.5 h before or at 0, 0.5, 1, 2, 4, 6, and 8 h after the addition of H5N1/QH PsV. The inhibitory activity of GRFT or GL25E on virus infection was determined as described above. In the washout assay, MDCK cells were incubated with GRFT or GL25E at 10 µmol/liter at 37°C for 1 h. Cells were then washed with PBS to remove unbound GRFT or GL25E, followed by the addition of H5N1/QH PsVs. In the control group, the cells preincubated with GRFT or GL25E were not washed before the addition of virus. After 12 h, the medium was changed, and luciferase activity was measured as described above.

### Inhibition of GRFT or GL25E on IAV and SARS-CoV-2 co-infection

To assess the inhibitory activity of GRFT or GL25E (EK1 as a control) on IAV PsV and SARS-CoV-2 PsV co-infection, A549 cells and Caco-2 cells were mixed at a 1:1 ratio and seeded in 96-well plates at 1 × 10^4^/well, while Calu-3 cells alone were seeded at 2 × 10^4^/well. After 24 h, a mixture of IAV H5N1/Thailand PsV and SARS-CoV-2 D614G PsV at a 1:1 ratio was added to the cells in the presence or absence of GRFT or GL25E. The inhibitory activity of GRFT, GL25E or EK1 on IAV and SARS-CoV-2 PsV infection was detected as described above.

### Immunoblotting and immunofluorescence assays

Immunoblotting and immunofluorescence analysis were performed as previously (46, 53). Briefly, MDCK, A549, and Calu-3 cells were seeded in wells of 6-well plates and 96-well plates, respectively. Followed by the addition of the mixture IAV at 0.2 MOI and an inhibitor at the indicated concentrations. For the immunoblotting, cells were lysed in 1 × RIPA buffer after washing with PBS. The protein concentration of total cell lysate was measured with BCA kit (Beyotime, Shanghai, China), separated via 12% SDS-PAGE, then and subjected to Western blot analysis. Commercial antibodies used for detecting IAV nucleoprotein (NP) included a rabbit polyclonal antibody against IAV NP (GeneTex, Irvine, CA, USA), a murine HRP-conjugated GAPDH monoclonal antibody (Proteintech, Wuhan, China), and an HRP-conjugated goat-anti-rabbit IgG (Dako, Copenhagen, Denmark). Image development was performed using an ECL Substrate kit (Tanon, Shanghai, China). For the immunofluorescence assay, cells were fixed and incubated with a primary antibody against IAV NP. A secondary antibody, goat-anti-rabbit IgG Alexa Fluor 488 (Abcam, Cambridge, UK), and DAPI (Invitrogen) were added sequentially according to the standard protocol. Cell imaging was observed and photographed using a fluorescence microscope (Invitrogen).

### Inhibition of authentic SARS-CoV-2 Omicron variants

Briefly, Caco-2 and Vero-E6 cells were used as target cells for RT-qPCR (54) and plaque-reduction assays (53), respectively. Cells were seeded in wells of a 96-well plate for 24 h before the addition of BA.2.2 or BA.5 variants (100 TCID_50_) in the presence or absence of GRFT, GL25E, or EK1 at indicated concentrations for 30 min. Supernatants were collected from Caco-2 cell culture after 48 h for RT-qPCR analysis using specific primers of nucleoprotein (N) genes, while Vero-E6 cells were stained with rabbit anti-SARS-CoV-2 N antibody and goat-anti-rabbit IgG Alexa Fluor 488. Plaques were visualized and calculated using a CTL ImmunoSpot S6 Ultra-V analyzer.

### Assays for cytotoxicity *in vitro* and safety *in vivo*

The CCK-8 kit (Dojindo, Kumamoto, Japan) was used to determine the cytotoxicity of GL25E. In brief, GL25E at a serial dilution was mixed with various cell lines (MDCK, A549, Calu-3, Caco-2, and Vero-E6) seeded in wells of 96-well plates. After 48 h, 10-fold diluted CCK-8 solution was added and incubated for 4 h. The absorbance at 450 nm was measured using a microplate reader (Tecan, USA).

The *in vivo* safety of GL25E in mice was assessed as previously described (32). Briefly, 8-week-old female C57BL/6J mice were randomly divided into two groups (*n* = 6) and received intranasal administration of PBS or a single-dose of GL25E diluted in PBS (10 mg/kg). Body weight changes were monitored for the next 2 weeks. ALT and creatinine levels in the serum of each group were measured using ALT and creatinine assay kits (NJJCBIO, Nanjing, China) at days 0, 1, 3, 5, and 7. After 30 days of administration, the mice in each group were euthanized, and their lungs, livers, and kidneys were collected for H&E staining.

### *In vivo* fluorescence imaging

GL25E protein was labeled with NHS ester following instructions in the manual of APExBIO (Houston, TX, USA). Eight-week-old female C57BL/6J mice were randomly divided into two groups. The first group (*n* = 6) received 10 mg/kg of Cy5-GL25E in PBS through nasal administration, while the second group (*n* = 3) received PBS as a control for measuring background fluorescence. The distribution of Cy5-GL25E was monitored at different time points (3, 24, 48, 72, and 96 h) using the IVIS Lumina K Series III *in vivo* imaging system (PerkinElmer, Waltham, MA, USA). After 48 h, mice were euthanized using isoflurane inhalation, and their lungs, livers, kidneys, spleens, and hearts were obtained for imaging (*n* = 3). The relevant radiant efficiency (Ps^−1^ cm^−2^ sr^−1^) (μW^−1^ cm^2^) was calculated using Living Image 4.4 software (55).

### Statistical analysis

Data were analyzed using GraphPad Prism 8.0, and the results were presented as the mean ± standard deviation. Statistical analysis was conducted using GraphPad Prism 8.0 with one-way ANOVA and a Neuman-Keuls *post hoc* analysis or Student’s *t*-test, depending on the situation. Statistical significance was defined as *P* < 0.05.

## SUPPLEMENTAL MATERIAL

Supplemental material mbio.00741-24-s0001.docx Fig. S1 to S3 and Tables S1 to S3.

ASM does not own the copyrights to Supplemental Material that may be linked to, or accessed through, an article. The authors have granted ASM a non-exclusive, world-wide license to publish the Supplemental Material files. Please contact the corresponding author directly for reuse.
