# Supplementary material for: Inhibition of influenza A virus and SARS-CoV-2 infection or co-infection by griffithsin and griffithsin-based bivalent entry inhibitor
Source: mBio. 2024 Apr 9;15(5):e00741-24. doi: 10.1128/mbio.00741-24 (PMC11077956; doi:10.1128/mbio.00741-24)
Supplement: Supplemental material — Fig. S1 to S3 and Tables S1 to S3. [file mbio.00741-24-s0001.docx]

**Supplemental materials**


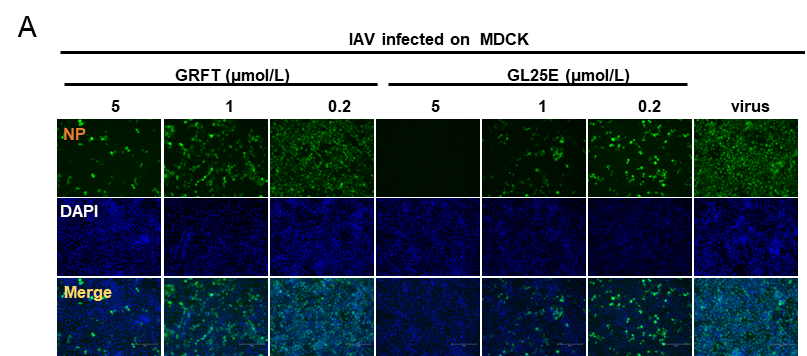


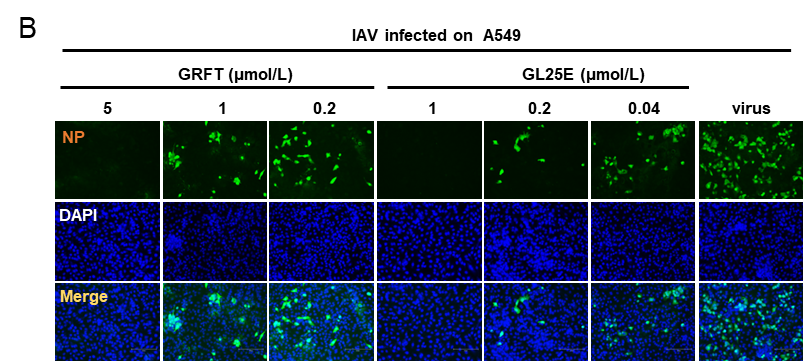


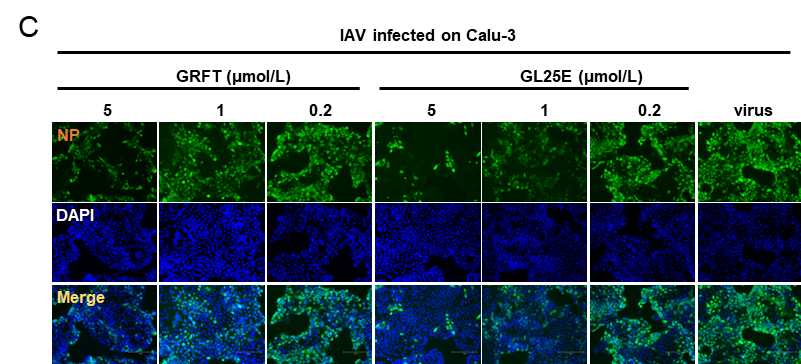


**Fig S1** Inhibition of authentic IAV infection by GRFT and GL25E as determined by indirect immunofluorescence assay. MDCK (A), A549 (B), and Calu-3 cells (C) were infected with A/WSN/33(H1N1) viruses (MOI=0.2) for 2 h in the presence or absence of GRFT and GL25E at the indicated concentration. Afterward, the medium was replaced with DMEM containing 2% FBS. After incubation at 37℃ for 12 h, the expression of viral NP was detected on the influenza virus–infected cells treated with GRFT and GL25E using the indirect immunofluorescence assay. The cells were stained with a rabbit polyclonal antibody against Influenza A virus Nucleoprotein (NP) for 3 h, followed by staining with Goat polyclonal to Rabbit IgG Alexa Fluor 488 for 1 hour. Nuclei were stained with DAPI. Scale bars: 150 μm.


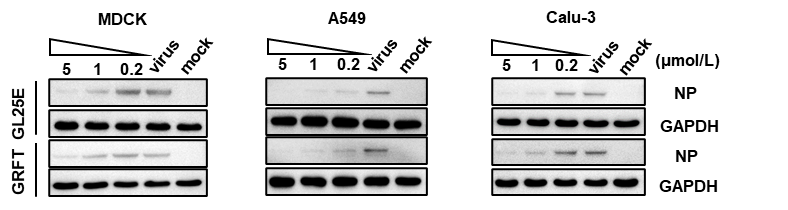


**Fig S2** Inhibition of authentic IAV infection by GRFT and GL25E as determined by Western blot. MDCK, A549, and Calu-3 cells were infected with A/WSN/33(H1N1) viruses (MOI=0.2) for 2 h in the presence or absence of GRFT and GL25E at the indicated concentration. Afterward, the medium was replaced with DMEM containing 2% FBS. After incubation at 37℃ for 12 h, cells were lysed and the protein expression of viral NP and GAPDH were detected via Western blot assay.

**
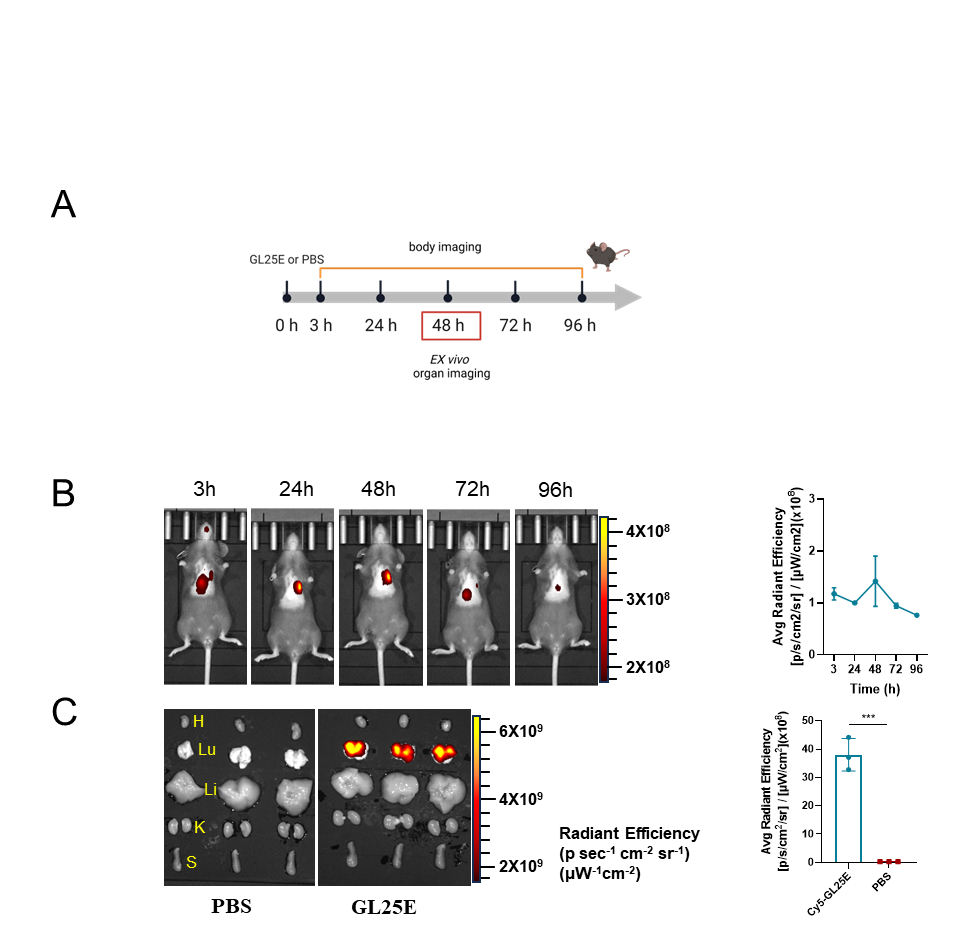
**

**Fig S3** In vivo duration and distribution of GL25E following inhalation administration. (A) Schematic diagram illustrating the biodistribution of GL25E in mice via intransnasal administration. (B) Bio-imaging of mouse body at various time points (left) and fluorescence intensity of Average Radiant Efficiency (right, n=2). (C) Mice were sacrificed at 48 h post GL25E administration and the dissected organs were imaged (n=3). Lu, Lung; K, Kidney; H, Heart; S, Spleen; Li, Liver (left) and quantification of lung tissue fluorescence signal (right). Statistical analysis was performed and analyzed using one-way ANOVA. **P*<0.05; ***P*<0.01; ****P*<0.001; *****P*<0.0001.

**Supplementary Table 1:** Histological evaluation scores of lung tissue in mice infected with IAV and treated with PBS, GRFT, or GL25E in prophylactic experiment.

| Group | Alveolar  edema | Hemorrhage | Neutrophilic  infiltration | Hyaline  membranes | Thickness of the alveolar wall | Atelectasis | Total |
| --- | --- | --- | --- | --- | --- | --- | --- |
| PBS | 0 | 1 | 3 | 0 | 3 | 3 | 10 |
| PBS | 0 | 1 | 2 | 0 | 3 | 2 | 8 |
| PBS | 0 | 1 | 3 | 0 | 2 | 2 | 8 |
| Mock | 0 | 0 | 1 | 0 | 1 | 0 | 2 |
| Mock | 0 | 0 | 0 | 0 | 1 | 0 | 1 |
| Mock | 0 | 0 | 0 | 0 | 1 | 0 | 1 |
| GRFT | 0 | 0 | 2 | 0 | 1 | 0 | 3 |
| GRFT | 0 | 0 | 0 | 0 | 1 | 0 | 1 |
| GRFT | 0 | 1 | 0 | 0 | 1 | 0 | 2 |
| GL25E | 0 | 0 | 1 | 0 | 1 | 0 | 2 |
| GL25E | 0 | 1 | 1 | 0 | 1 | 0 | 3 |
| GL25E | 0 | 0 | 1 | 0 | 1 | 0 | 2 |

**Supplementary Table 2:** Inhibitory activity of GRFT and GL25E against infection by authentic SARS-CoV-2 Omicron BA.2.2 and BA.5 strains detected using the plaque-reduction assay.

| Compound | IC_50_ (nmol/L) | |
| --- | --- | --- |
|  | BA.2.2 | BA.5 |
| GRFT | 100.9 | 123.7 |
| GL25E | 94.1 | 63.2 |
| *P* | 0.949 | 0.747 |

Statistical analysis was performed and calculated by GraphPad Prism 8.0.

**Supplementary Table 3:** The primer sequences used for qRT-PCR are as follows.

| Primer | Sequence 5’ - 3’ |
| --- | --- |
| *HA* forward | TTCCCAAGATCCATCCGGCAA |
| *HA* reverse | CCTGCTCGAAGACAGCCACAACG |
| *GAPDH* forward | AGGGCAATGCCAGCCCCAGCG |
| *GAPDH* reverse | AGGCGTCGGAGGGCCCCCTC |
| *HA1* forward | GATCGATATCGATGGAGAAAATAGTGCTTCTTCTT |
| *HA1* reverse | CATGCCATGGCTCTTCTCTCTCCTTGAGGGCTA |
| *SARS-CoV-2-N* Taqman probe | FAM-TTGCTGCTGCTTGACAGATT-TAMRA |
| *SARS-CoV-2-N* forward | GGGGAACTTCTCCTGCTAGAAT |
| *SARS-CoV-2-N* reverse | CAGACATTTTGCTCTCAAGCTG |
